# A hierarchical loss and its problems when classifying non-hierarchically

**DOI:** 10.1371/journal.pone.0226222

**Published:** 2019-12-19

**Authors:** Cinna Wu, Mark Tygert, Yann LeCun

**Affiliations:** 1 Facebook, Menlo Park, CA, United States of America; 2 Facebook, New York, NY, United States of America; Ulm University, GERMANY

## Abstract

Failing to distinguish between a sheepdog and a skyscraper should be worse and penalized more than failing to distinguish between a sheepdog and a poodle; after all, sheepdogs and poodles are both breeds of dogs. However, existing metrics of failure (so-called “loss” or “win”) used in textual or visual classification/recognition via neural networks seldom leverage a-priori information, such as a sheepdog being more similar to a poodle than to a skyscraper. We define a metric that, inter alia, can penalize failure to distinguish between a sheepdog and a skyscraper more than failure to distinguish between a sheepdog and a poodle. Unlike previously employed possibilities, this metric is based on an ultrametric tree associated with any given tree organization into a semantically meaningful hierarchy of a classifier’s classes. An ultrametric tree is a tree with a so-called ultrametric distance metric such that all leaves are at the same distance from the root. Unfortunately, extensive numerical experiments indicate that the standard practice of training neural networks via stochastic gradient descent with random starting points often drives down the hierarchical loss nearly as much when minimizing the standard cross-entropy loss as when trying to minimize the hierarchical loss directly. Thus, this hierarchical loss is unreliable as an objective for plain, randomly started stochastic gradient descent to minimize; the main value of the hierarchical loss may be merely as a meaningful metric of success of a classifier.

## Introduction

Metrics for classifier accuracy used in the neural network methods of [1] seldom account for semantically meaningful organizations of the classes; these metrics neglect, for instance, that sheepdogs and poodles are dogs, that dogs and cats are mammals, that mammals, birds, reptiles, amphibians, and fish are vertebrates, and so on. Below, we define a metric—the amount of the “win” or “winnings” for a classification—that accounts for a given organization of the classes into a tree. During an optimization (also known as “training”), we want to maximize the win or, equivalently, minimize the “loss” (loss is the negative of the win). We contrast the hierarchical win to the existing universal standard, the cross-entropy loss discussed by [1] (and by many others). We caution that several of our experiments indicate that plain stochastic gradient descent optimization with random starting points can get stuck in local optima; even so, the hierarchical win can serve as a good metric of success or figure of merit for the accuracy of the classifier.

The approach detailed below is a special case of the general methods of [2], [3], and their references. The particular special cases discussed by them and by [4–9] allocate different weights to different leaves, not leveraging “ultrametric trees” as in the present paper (an ultrametric tree is a tree with a so-called ultrametric distance metric such that all leaves are at the same distance from the root, as in the phylogenetics discussed, for example, by [10]). The distinguishing characteristic of the distance metric constructed below is that all leaves are at the same distance from the root, as in an ultrametric tree; this provides a definition and measure of successful classification that is different from other options.

A related topic is hierarchical classification, as reviewed by [11] (with further use more recently by [12] and [13], for example); however, the present paper considers only classification into a given hierarchy’s finest-level classes. The design options for classification into only the finest-level classes are more circumscribed, yet such classification is easier to use, implement, and interface with existing codes. The existing standard cross-entropy is the chief alternative in the setting of the present paper (that is, for classification into only the finest-level classes). Cross-entropy loss is the negative of the logarithm of our hierarchical win when the hierarchy is “flat,” that is, when the hierarchy is the degenerate case in which all classes are leaves attached to the same root. Extensive experiments demonstrate the advantages of hierarchical loss in comparison to the conventional cross-entropy.

Hierarchical classification, such as that reviewed by [11], is more sophisticated (and potentially more powerful) than what the present article considers. The present paper only changes the metric of success (and objective function), not altering the procedure for classification—hierarchical procedures for classification are often avoided due to complications of systems development and systems programming. We were tasked with changing the figure of merit for measuring success of a classifier without being allowed to pursue more ambitious, full-blown hierarchical processes for classification. The actual real-world motivation for the hierarchical loss of the present article was in recommending places to users, where (for example) sending users to a Vietnamese restaurant when they request a Thai restaurant should be penalized less than sending them to a park instead—different kinds of restaurants are more similar to each other than to a park.

We warn that minimizing the hierarchical loss directly via the usual stochastic gradient descent with random starting points was often fruitless even in relatively simple numerical experiments reported below. The complexity of systems deployed in practice typically makes hierarchical procedures for optimization very hard to implement, yet altering only the objective of the optimization has frustratingly little effect on the hierarchical loss during several tests in Section *Results and discussion* below.

The remainder of the present paper has the following structure: Section *Methods* constructs the hierarchical loss and win. Section *Results and discussion* illustrates and evaluates the hierarchical loss and win via several numerical experiments. Section *Conclusion* draws conclusions and proposes directions for future work.

## Methods

Concretely, suppose that we want to classify each input into one of many classes, and that these classes are the leaves in a tree which organizes them into a semantically meaningful hierarchy. Suppose further that a classifier maps an input to an output probability distribution over the leaves, hopefully concentrated on the leaf corresponding to the correct class for the input. We define the probability of any node in the tree to be the sum of the probabilities of all leaves falling under the node (the node represents an aggregated class consisting of all these leaves); a leaf falls under the node if the node is on the path from the root to the leaf. We then define the amount of the “win” or “winnings” to be the weighted sum (with weights as detailed shortly) of the probabilities of the nodes along the path from the root to the leaf corresponding to the correct class.

To calculate the win, we sum across all nodes on the path from the root to the leaf corresponding to the correct class, including both the root and the leaf, weighting the probability at the first node (that is, at the root) by 1/2, weighting at the second node by 1/2^2^, weighting at the third node by 1/2^3^, weighting at the fourth node by 1/2^4^, and so on until the final leaf. We then add to this sum the probability at the final leaf, weighted with the same weight as in the sum, that is, we double-count the final leaf. We justify the double-counting shortly.

To compute the probability of each node given the probability distribution over the leaves, we propagate the leaf probabilities through the tree as follows. We begin by storing a zero at each node in the tree. Then, for each leaf, we add the probability associated with the leaf to the value stored at each node on the path from the root to the leaf, including at the root and at the leaf. We save these accumulated values as the propagated probabilities (storing the value 1 at the root—the sum of all the probabilities is 1 of course).

Thus, if the probability of the final leaf is 1, then the win is 1. The win can be as large as 1 (this happens when the classification is completely certain and correct) or as small as 1/2 (this happens when the classification is as wrong as possible). The win being 1 whenever the probability of the final leaf is 1—irrespective of which is the final leaf—means that the weights form an “ultrametric tree,” as in the phylogenetics discussed, for example, by [10]. This justifies double-counting the final leaf.

**Fig 1:**
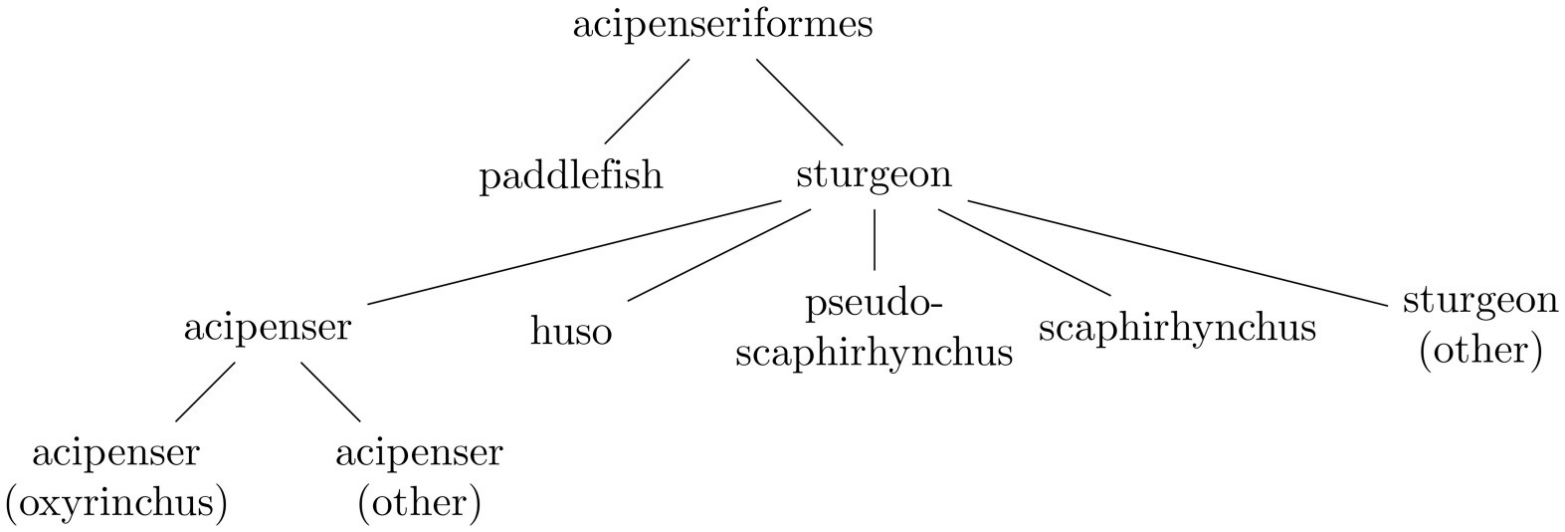
This is a hierarchy of types of fish (but not the whole hierarchy from Subsection *DBpedia fish* of Section *Results and discussion*).

**Fig 2:**
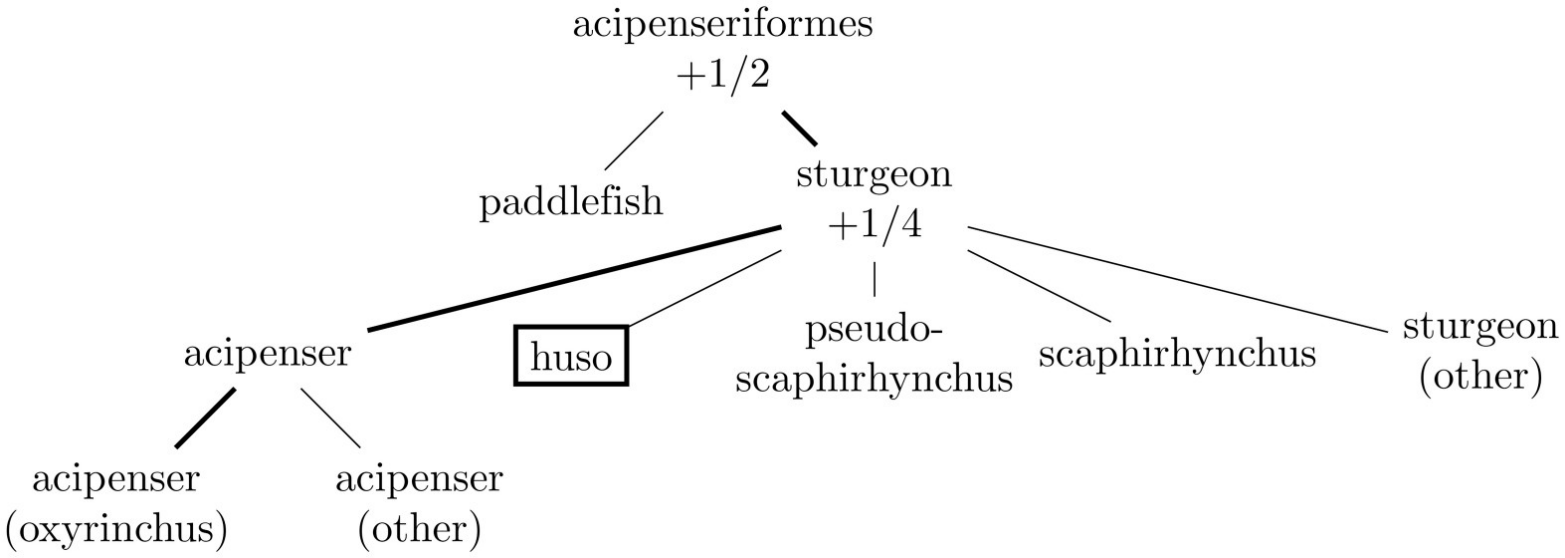
This depicts the hierarchy of Fig 1 when the correct target leaf is “acipenser (oxyrinchus)” but the leaf predicted with probability 1 (the “single best class” of Subsection *Choosing a single best class*) is “huso” (as indicated with a box around “huso”). In this case, the hierarchical win is 1/2 + 1/4 = 3/4, as “acipenseriformes” and “sturgeon” are the nodes on both the path from the root “acipenseriformes” to “acipenser (oxyrinchus)” and the path from the root “acipenseriformes” to “huso”.

**Fig 3:**
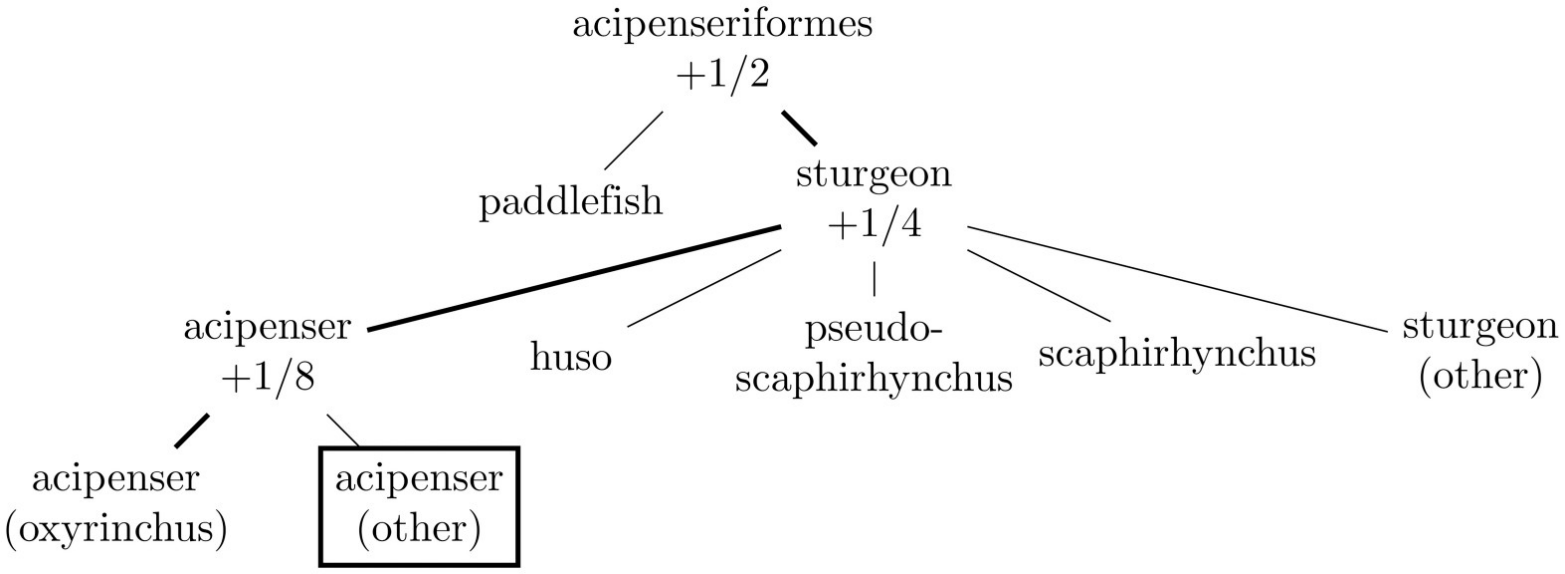
This depicts the hierarchy of Fig 1 when the correct target leaf is “acipenser (oxyrinchus)” but the leaf predicted with probability 1 (the “single best class” of Subsection *Choosing a single best class*) is “acipenser (other)” (as indicated with a box around “acipenser (other)”). In this case, the hierarchical win is 1/2 + 1/4 + 1/8 = 7/8, as “acipenseriformes,” “sturgeon,” and “acipenser” are the nodes on both the path from the root “acipenseriformes” to “acipenser (oxyrinchus)” and the path from the root “acipenseriformes” to “acipenser (other)”.

**Fig 4:**
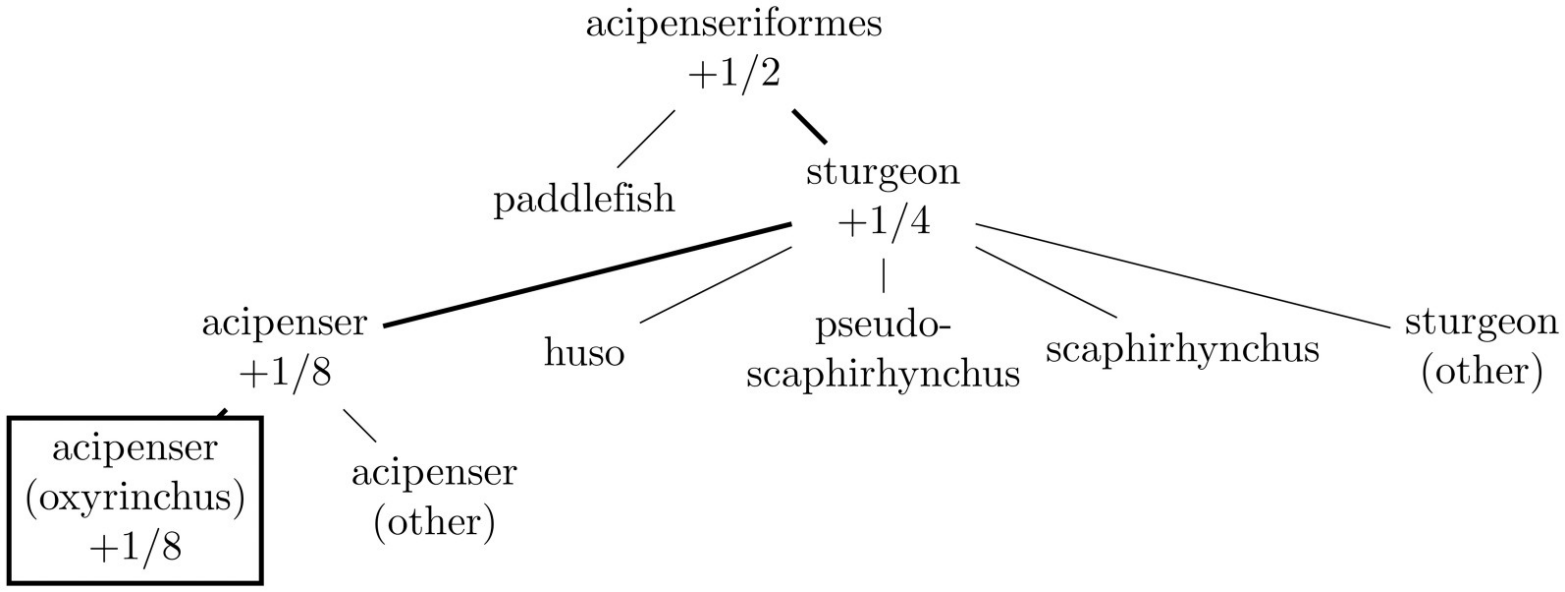
This depicts the hierarchy of Fig 1 when the correct target leaf is “acipenser (oxyrinchus),” the same as the leaf predicted with probability 1 (the “single best class” of Subsection *Choosing a single best class*). In this case, the hierarchical win is 1/2 + 1/4 + 1/8 + 1/8 = 1 (as with all correct predictions); the final node is double-counted at 1/8 = 1/16 + 1/16.

Figs 1–4 illustrate the evaluation of the hierarchical win via examples. The following two subsections summarize in pseudocode the algorithms for propagating the probabilities and for calculating the win, respectively (the latter algorithm runs the former as its initial step).

### An algorithm for propagating probabilities

**Input**: a discrete probability distribution over the leaves of a tree

**Output**: a scalar value (the total probability) at each node in the tree

**Procedure**:

**Store** the value 0 at each node in the tree.

**For** each leaf,

 **for** each node in the path from the root to the leaf (including both the root and the leaf),

  **add** to the value stored at the node the probability of the leaf.

### An algorithm for computing the “win” or “winnings”

**Input**: two inputs, namely (1) a discrete probability distribution over the leaves of a tree, and (2) which leaf corresponds to a completely correct classification

**Output**: a single scalar value (the “win” or “winnings”)

**Procedure**:

**Run** *An algorithm for propagating probabilities* to obtain a scalar value at each node in the tree.

**Store** the value 0 in an accumulator.

**Define** *ℓ* to be the number of nodes in the path from the root (node 1) to the leaf (node *ℓ*).

**For** *j* = 1, 2, …, *ℓ*,

 **add** to the accumulated value 2^−*j*^ times the value stored at the path’s *j*th node.

**Add** to the accumulated value 2^−*ℓ*^ times the value stored at the path’s *ℓ*th node (the leaf).

**Return** the final accumulated value.

### Calculation of gradients

To facilitate optimization via gradient-based methods, we now detail how to compute the gradient of the hierarchical win with respect to the input distribution: Relaxing the constraint that the distribution over the leaves be a probability distribution, that is, that the “probabilities” of the leaves be nonnegative and sum to 1, the algorithms specified in the preceding two subsections yield a value for the win as a function of any distribution over the leaves. Without the constraint that the distribution over the leaves be a probability distribution, the win is actually a linear function of the distribution, that is, the win is the dot product between the input distribution and another vector (where this other vector depends on which leaf corresponds to the correct classification); the gradient of the win with respect to the input is therefore just the vector in the dot product. An entry of this gradient vector, say the *j*th entry, is equal to the win for the distribution over the leaves that consists of all zeros except for one value of one on the *j*th leaf; this win is equal to the sum of the series 1/2+ 1/2^2^ + 1/2^3^ + 1/2^4^ + …, truncated to the number of terms equal to the number of nodes for which the path from the root to the correct leaf and the path from the root to the *j*th leaf coincide (or not truncated at all if the *j*th leaf happens to be the same as the leaf for a correct classification).

### Choosing a single best class

If forced to choose a single class corresponding to a leaf of the given hierarchy (rather than classifying into a probability distribution over all leaves) for the final output of the classification, we first identify the node having greatest probability among all nodes (including leaves) at the coarsest level, then the node having greatest probability among all nodes (including leaves) falling under the first node selected, then the node having greatest probability among all nodes (including leaves) falling under the second node selected, and so on, until we select a leaf. The leaf we select corresponds to the class we choose. The following subsection summarizes in pseudocode this procedure for selecting a single best leaf.

### An algorithm for choosing the single best leaf

**Input**: a discrete probability distribution over the leaves of a tree

**Output**: a single leaf of the tree

**Procedure**:

**Run** *An algorithm for propagating probabilities* to obtain a scalar value at each node in the tree.

**Move** to the root.

**Repeating until** at a leaf,

 **follow** (to the next finer level) the branch containing the greatest among all scalar values stored at this next finer level in the current subtree.

**Return** the final leaf.

### Logarithms when using a softmax or independent samples

In order to provide appropriately normalized results, the input to the hierarchical loss needs to be a (discrete) probability distribution, not just an arbitrary collection of numbers. A “softmax” provides a good, standard means of converting any collection of real numbers into a proper probability distribution. Recall that the softmax of a sequence *x*_1_, *x*_2_, …, *x*_*n*_ of *n* numbers is the normalized sequence exp(*x*_1_)/*Z*, exp(*x*_2_)/*Z*, …, exp(*x*_*n*_)/*Z*, where $Z=\sum_{k=1}^{n}\text{exp}\left(x_{k}\right)$. Notice that each of the normalized numbers lies between 0 and 1, and the sum of the numbers in the normalized sequence is 1—the normalized sequence is a proper probability distribution.

When generating the probability distribution over the leaves via a softmax, we should optimize based on the logarithm of the “win” introduced in the subsections above rather than the “win” itself. In this case, omitting the contribution of the root to the objective value and its gradient makes the most sense, ensuring that a flat hierarchy (that is, a hierarchy which has only one level aside from the root’s) results in the same training as with the usual cross-entropy loss. Taking the logarithm also makes sense because the joint probability of stochastically independent samples is the product of the probabilities of the individual samples, making averaging across the different samples the logarithm of a function (the function could be the win) make more sense than averaging the function directly. That said, taking the logarithm emphasizes highly misclassified samples, which may not be desirable if misclassifying a few samples (while simultaneously reporting high confidence in their classification) should be acceptable.

Indeed, if the logarithm of the win for even a single sample is infinite, then the average of the logarithm of the win is also infinite, irrespective of the values for other samples. Whether the hierarchy is full or flat, training on the logarithms of wins is very stringent, whereas the wins without the logarithms can be more meaningful as metrics of success or figures of merit. It can make good sense to train on the logarithm, which works really hard to accommodate and learn from the samples which are hardest to classify correctly, but to make the metric of success or figure of merit be robust against such uninteresting outliers. Thus, training with the logarithm of the win can make good sense, where the win—without the logarithm—is the metric of success or figure of merit for the testing or validation stage.

With or without a logarithm, we henceforth omit the contribution of the root to the hierarchical wins and losses that we report, and we multiply by 2 the resulting win, so that its minimal and maximal possible values become 0 and 1 (with 0 corresponding to the most incorrect possible classification, and with 1 corresponding to the completely correct classification).

## Results and discussion

We illustrate the hierarchical loss and its performance using supervised learning for text classification with fastText of [14]. The tables report performance across several experiments, with the columns “training loss (rate, epochs)” listing the following three parameters for training: (1) the form of the loss function used during training (as explained shortly), (2) the initial learning rate which tapers linearly to 0 over the course of training, and (3) the total number of sweeps through the data performed during training (too many sweeps results in overfitting). The training loss “flat” refers to training using the existing standard cross-entropy loss (that is invariably the default in machine learning), which is the same as the negative of the natural logarithm of the hierarchical win when using a flat hierarchy in which all labelable classes are leaves attached to the root (as discussed in Subsection *Logarithms when using a softmax or independent samples* of Section *Methods*). The training loss “raw” refers to training using the hierarchical loss, using the full hierarchy. The training loss “log” refers to training using the negative of the natural logarithm of the hierarchical win, using the full hierarchy. The training loss “coarse” refers to training using the usual cross-entropy loss for classification into only the coarsest (aggregated) classes in the hierarchy (based on a suitably smaller softmax for input to the loss). The values reported in the tables for the learning rate and number of epochs yielded among the best results for the accuracies discussed in the following paragraphs, during a limited grid search around the reported values (if increasing the epochs beyond the reported value had little effect, then we report the smaller number of epochs); the appendix details the settings considered.

The columns “one-hot win via hierarchy” display the average over all testing samples of the hierarchical win fed with the results of a one-hot encoding of the class chosen according to Subsection *Choosing a single best class* of Section *Methods*. (The one-hot encoding of a class is the vector whose entries are all zeros aside from a single entry of one in the position corresponding to the class.) The columns “softmax win via hierarchy” display the average over all testing samples of the hierarchical win fed with the results of a softmax from fastText of [14] (Subsection *Logarithms when using a softmax or independent samples* of Section *Methods* above reviews the definition of “softmax”). The columns “−log of win via hierarchy” display the average over all testing samples of the negative of the natural logarithm of the hierarchical win fed with the results of a softmax from fastText (Subsection *Logarithms when using a softmax or independent samples* of Section *Methods* above reviews the definition of “softmax”). The columns “cross-entropy” display the average over all testing samples of the usual cross-entropy loss, which is the same as the negative of the natural logarithm of the hierarchical win fed with the results of a softmax when using a “flat” hierarchy in which all labelable classes are leaves attached to the root (as discussed in Subsection *Logarithms when using a softmax or independent samples* of Section *Methods*). Please note that the hierarchy and form of the training loss may be different than the hierarchies and forms of the testing losses and wins; for example, using the “flat” training loss does not alter the full hierarchy used in the “−log of win via hierarchy.”

The columns “coarsest accuracy” display the fraction of testing samples for which the coarsest classes containing the fine classes chosen during the classification are correct, when classifying each sample into exactly one class, as in Subsection *Choosing a single best class* of Section *Methods*. The columns “parents’ accuracy” display the fraction of testing samples for which the parents of the classes chosen during the classification are correct, when classifying each sample into exactly one class; the parents are the same as the coarsest classes in Tables 3 and 4, as the experiments reported in Tables 3 and 4 pertain to hierarchies with only two levels (excluding the root). The columns “finest accuracy” display the fraction of testing samples classified correctly, when classifying each into exactly one finest-level class, again as in Subsection *Choosing a single best class* of Section *Methods*.

The columns “aggregate precision,” “aggregate recall,” and “aggregate F1” refer to the hierarchical precision, hierarchical recall, and hierarchical F score detailed in the survey of [11]. The columns “aggregate precision” display the total number of nodes on the path from the root to the class chosen when classifying each sample into exactly one class, as in Subsection *Choosing a single best class* of Section *Methods*, that are also on the path from the root to the correct class, divided by the total number of nodes on the path from the root to the chosen class (aggregating both totals separately over all samples). The columns “aggregate recall” display the same total as for the aggregate precision, but divided by the total number of nodes on the path from the root to the correct class, rather than on the path from the root to the chosen class (while again aggregating both totals in the quotient separately over all samples). The columns “aggregate F1” display the harmonic mean of the aggregate precision and the aggregate recall. When counting these total numbers of nodes, we never count the root node, but always count any leaf node on the paths. Since the aggregation in the quotients defining these metrics happens separately for each numerator and denominator, these aggregate metrics are not mean values averaged over the samples, unlike all other quantities reported in the tables. As elaborated by [11], these aggregate metrics are common in the study of hierarchical classification (however, hierarchical classification is beyond the scope of the present paper, for the reasons outlined in Section *Introduction*; the present paper reports the aggregate metrics at the request of some readers, purely for reference and comparative purposes).

The last lines of the tables remind the reader that the best classifier would have

- higher one-hot win via hierarchy
- higher softmax win via hierarchy
- lower −log of win via hierarchy
- lower cross-entropy
- higher coarsest accuracy
- higher parents’ accuracy
- higher finest accuracy
- higher aggregate precision
- higher aggregate recall
- higher aggregate F1

We follow the recommendation from Subsection *Logarithms when using a softmax or independent samples* of Section *Methods* above, maximizing the hierarchical win (or its logarithm) calculated without any contribution from the root (excluding the root makes a small difference when taking the logarithm); please note the very last paragraph in the preceeding section—the hierarchical wins reported here omit the contribution from the root and rescale the result by a factor of 2.

We hashed bigrams (pairs of words) into a million buckets and trained using stochastic gradient descent, setting the learning rate to start at the values indicated in the tables (these worked at least as well as other settings), then decaying linearly to 0 over the course of training, as done by [14]; the starting point for the stochastic gradient descent was random. The “learning rate” is also known as the “step size” or “step length” in the update for each iteration (step) of stochastic gradient descent.

Our implementation couples the C++ software of [14] with a Python prototype. Industrial deployment requires acceleration of the Python prototype (rewriting in C++, for instance), but our tests are sufficient for estimating the ensuing gains in accuracy and illustrating the figure of merit, providing a proof of principle. In particular, our experiments indicate that the gains in accuracy due to training with the hierarchical loss are meager except in special circumstances detailed in the subsections below and summarized in the conclusion. Pending further development as suggested in the conclusion, the main present use for the hierarchical win should be as a metric of success or figure of merit—a good notion of “accuracy”—at least when training with plain stochastic gradient descent coupled to backpropagation.

Several of our datasets consider the setting in which each class includes at most one training sample. Such a setting is a proxy for applications in “open-world classification,” “few-shot learning,” and “personalization,” which often involves limited or even (for many classes) no training data per individual class, though there may be many individuals. Access to a hierarchy of the classes enables meaningful classification even for classes not represented in the training data, as the hierarchy itself indicates that some classes are very much like others (some of which may appear in the training data). Applying a hierarchical loss is perhaps the simplest method for effective classification and quantification of accuracy when there is at most one training sample per class.

When reading the following subsections, please recall that the standard cross-entropy loss is the same as the negative of the logarithm of the hierarchical win with a flat hierarchy.

The following subsections detail our experiments and datasets; the subsequent section, Section *Conclusion*, elucidates the consequences of these experiments. For the experiments, we used the hierarchies that accompanied the datasets; all are available at the web links (URLs) given in the following subsections. Two datasets—those in Subsections *Yahoo Answers* and *DBpedia*—provided no hierarchy, so for these we constructed the hierarchies described below in Subsections *Yahoo Answers* and *DBpedia*. In all cases, the results display little dependence on the dimension of the space into which we embedded words and their bigrams (words are unigrams), in accordance with [14], provided only that the dimension is somewhat larger than the number of classes for targets in the classification.

### RCV1-v2

Table 1 reports results on RCV1-v2 of [15], which is available for download at http://jmlr.csail.mit.edu/papers/volume5/lewis04a. This dataset includes a hierarchy of 364 classes (semantically, these are classes of industries); each sample from the dataset comes associated with at least one of these 364 class labels, whether or not the class is an internal node of the tree or a leaf. Each sample from the dataset consists of filtered, tokenized text from Reuters news articles (“article” means the title and body text). As described by [15], labels associated with internal nodes in the original hierarchy may be viewed as leaves that fall under those internal nodes while not classifying into any of the lower-level nodes. In our hierarchy, we hence duplicate every internal node into an “other” class under that node, such that the “other” class is a leaf.

**Table 1:** Results on RCV1-v2, tested on 5,000 samples.

training loss (rate, epochs)	one-hot win via hierarchy	softmax win via hierarchy	−log of win via hierarchy	cross entropy
flat (2, 4)	.85	.80	.52	.95
raw (12, 4)	.51	.50	4.2	∞
log (4, 4)	.74	.72	.76	4.4
the ideal	higher	higher	lower	lower

training loss (rate, epochs)	coarsest accuracy	parents’ accuracy	finest accuracy
flat (2, 4)	.88	.82	.80
raw (12, 4)	.74	.26	.21
log (4, 4)	.87	.63	.53
coarse (.05, 100)	.88		
the ideal	higher	higher	higher

training loss (rate, epochs)	aggregate precision	aggregate recall	aggregate F1
flat (2, 4)	.84	.84	.84
raw (12, 4)	.38	.46	.42
log (4, 4)	.67	.70	.69
the ideal	higher	higher	higher

We discard every sample from the dataset associated with more than one label, and swap the training and testing sets (since the original training set is small, whereas the original testing set is large). Furthermore, we randomly permute all samples in both the training and testing sets, and subsample to 5,000 samples for testing and 200,000 for training. In the hierarchy, there are 10 coarsest classes and 61 parents of the 254 leaves that were actually represented by any samples in the training and testing sets. The space into which we embedded the words and their pairs (bigrams) was 1,000-dimensional. For training the classifier into only the coarsest (aggregated) classes, we embedded the words and bigrams into a 20-dimensional space.

For this dataset, optimizing based on the hierarchical loss (with or without a logarithm) yields worse accuracy according to all metrics considered compared to optimizing based on the standard cross-entropy loss.

### Subsampled RCV1-v2

Table 2 reports results on the same dataset RCV1-v2 (which is available for download at http://jmlr.csail.mit.edu/papers/volume5/lewis04a) of the preceeding subsection, but retaining only one training sample for each class label. The training set thus consists of 254 samples (many of the 364 possible class labels had no corresponding samples in the training set from the preceeding subsection). As in the preceding subsection, the hierarchy has 10 coarsest classes and 61 parents of the 254 leaves that were used in the training and testing sets. We again embedded the words and their bigrams into a 1,000-dimensional space, while for training the classifier into only the coarsest (aggregated) classes, we again used only 20 dimensions.

**Table 2:** Results on RCV1-v2 with at most one training sample per class, tested on 5,000 samples.

training loss (rate, epochs)	one-hot win via hierarchy	softmax win via hierarchy	−log of win via hierarchy	cross entropy
flat (.04, 9)	.14	.07	2.8	5.5
raw (45, 500)	.16	.15	7.2	13
log (3, 40)	.17	.09	2.7	5.5
the ideal	higher	higher	lower	lower

training loss (rate, epochs)	coarsest accuracy	parents’ accuracy	finest accuracy
flat (.04, 9)	.209	.086	.051
raw (45, 500)	.235	.095	.052
log (3, 40)	.258	.089	.064
coarse (4, 1000)	.324		
the ideal	higher	higher	higher

training loss (rate, epochs)	aggregate precision	aggregate recall	aggregate F1
flat (.04, 9)	.121	.118	.120
raw (45, 500)	.132	.148	.140
log (3, 40)	.141	.166	.153
the ideal	higher	higher	higher

For this subsampled RCV1-v2, optimizing based on the negative of the natural logarithm of the hierarchical win yields better accuracy according to all metrics considered compared to optimizing based on the standard cross-entropy loss, except on the negative of the natural logarithm of the hierarchical win and the cross-entropy loss (for which the accuracies are similar).

### Yahoo Answers

Table 3 reports results on the Yahoo Answers subset introduced by [16], which is available for download at http://goo.gl/JyCnZq. This dataset includes 10 classes (semantically, these are classes of interest groups); each sample from the dataset comes associated with exactly one of these 10 class labels. Each sample from the dataset consists of normalized text from questions and answers given on a website devoted to Q&A. The space into which we embedded the words and their bigrams was 20-dimensional, while for training the classifier into only the coarsest (aggregated) classes, we embedded the words and bigrams into a 10-dimensional space. For the nontrivial hierarchy, we grouped the 10 classes into 4 superclasses:

1. **Leisure**: Entertainment and Music, Society and Culture, and Sports
2. **Newsworthy**: Business and Finance, and Politics and Government
3. **Relations**: Family and Relationships
4. **Science and Technology**: Computer and Internet, Education and Reference, Health, and Science and Mathematics

**Table 3:** Results on Yahoo Answers, tested on 60,000 samples.

training loss (rate, epochs)	one-hot win via hierarchy	softmax win via hierarchy	−log of win via hierarchy	cross entropy
flat (.1, 4)	.76	.67	.67	.91
raw (1, 4)	.65	.64	2.6	∞
log (.1, 4)	.76	.67	.68	1.0
the ideal	higher	higher	lower	lower

training loss (rate, epochs)	coarsest accuracy	finest accuracy
flat (.1, 4)	.80	.72
raw (1, 4)	.79	.50
log (.1, 4)	.80	.71
coarse (.1, 4)	.80	
the ideal	higher	higher

training loss (rate, epochs)	aggregate precision	aggregate recall	aggregate F1
flat (.1, 4)	.76	.76	.76
raw (1, 4)	.64	.64	.64
log (.1, 4)	.76	.76	.76
the ideal	higher	higher	higher

With only 10 classes and two levels for the classification hierarchy, Table 3 indicates that training with or without the hierarchical loss makes little difference.

### DBpedia

Table 4 reports results on the DBpedia subset introduced by [16], which is available for download at http://goo.gl/JyCnZq. This dataset includes 14 classes (semantically, these are categories from DBpedia); each sample from the dataset comes associated with exactly one of these 14 class labels. Each sample from the dataset consists of normalized text from DBpedia articles (“article” means the title and body text). We embedded the words and their bigrams into a 20-dimensional space, except when training a classifier into only the coarsest (aggregated) classes, in which case a space with only 10 dimensions was fine. For the nontrivial hierarchy, we grouped the 14 classes into 6 superclasses:

1. **Institution**: Company and EducationalInstitution
2. **Man-made**: Building and MeansOfTransportation
3. **Media**: Album, Film, and WrittenWork
4. **Organism**: Animal and Plant
5. **Person**: Artist, Athlete, and OfficeHolder
6. **Place**: NaturalPlace and Village

**Table 4:** Results on DBpedia, tested on 70,000 samples.

training loss (rate, epochs)	one-hot win via hierarchy	softmax win via hierarchy	−log of win via hierarchy	cross entropy
flat (.5, 4)	.989	.986	.034	.054
raw (1, 4)	.813	.811	.312	6.06
log (.5, 4)	.988	.985	.036	.063
the ideal	higher	higher	lower	lower

training loss (rate, epochs)	coarsest accuracy	finest accuracy
flat (.5, 4)	.992	.986
raw (1, 4)	.989	.636
log (.5, 4)	.992	.985
coarse (.3, 4)	.992	
the ideal	higher	higher

training loss (rate, epochs)	aggregate precision	aggregate recall	aggregate F1
flat (.5, 4)	.989	.989	.989
raw (1, 4)	.813	.813	.813
log (.5, 4)	.988	.988	.988
the ideal	higher	higher	higher

With merely 14 classes and two levels for the classification hierarchy, Table 4 shows that training with or without the hierarchical loss makes little difference.

### DBpedia fish

Table 5 reports results on the subset corresponding to fish from the DBpedia of [17], which is available for download at http://web.informatik.uni-mannheim.de/DBpediaAsTables/DBpedia-3.9/json/Fish.json.gz. This dataset includes 1,298 classes (semantically, these are taxonomic groups of fish, such as species containing sub-species, genera containing species, or families containing genera—DBpedia extends to different depths of taxonomic rank for different kinds of fish; our classes are the parents of the leaves in the DBpedia tree). Each sample from the dataset consists of normalized text from the lead section (the introduction) of the Wikipedia article on the associated type of fish, with all sub-species, species, genus, family, and order names removed from the associated Wikipedia article (DBpedia derives from Wikipedia, as discussed by [17]). For each of our finest-level classes, we chose uniformly at random one leaf in the DBpedia taxonomic tree of fish to be a sample in the training set, reserving the other leaves for the testing set (the testing set consists of a random selection of 6,000 of these leaves). In the hierarchy, there are 94 coarsest classes, 367 parents of the leaves in our tree, and 1,298 leaves in our tree. Optimizing the hierarchical win—without any logarithm—was wholly ineffective, always resulting in assigning the same finest-level class to all input samples (with the particular class assigned varying according to the extent of training and the random starting point). So taking the logarithm of the hierarchical win was absolutely necessary to train successfully. For training the classifier into only the coarsest (aggregated) classes, we embedded the Wikipedia articles’ words and bigrams into a 200-dimensional space rather than the larger 2,000-dimensional space used for classifying into all 1,298 classes.

**Table 5:** Results on DBpedia fish, tested on 6,000 samples.

training loss (rate, epochs)	one-hot win via hierarchy	softmax win via hierarchy	−log of win via hierarchy	cross entropy
flat (5, 25)	.27	.25	4.4	16
raw (200, 200)	.17	.17	6.5	∞
log (5, 50)	.38	.36	3.5	19
the ideal	higher	higher	lower	lower

training loss (rate, epochs)	coarsest accuracy	parents’ accuracy	finest accuracy
flat (5, 25)	.38	.26	.065
raw (200, 200)	.31	.04	.001
log (5, 50)	.60	.22	.014
coarse (3, 70)	.60		
the ideal	higher	higher	higher

training loss (rate, epochs)	aggregate precision	aggregate recall	aggregate F1
flat (5, 25)	.24	.25	.24
raw (200, 200)	.12	.13	.12
log (5, 50)	.30	.33	.31
the ideal	higher	higher	higher

Here, optimizing based on the negative of the logarithm of the hierarchical win yields much better coarsest accuracy and hierarchical wins than optimizing based on the standard cross-entropy loss, while optimizing based on the standard cross-entropy loss yields much better finest accuracy and cross-entropy. When optimizing based on the negative of the natural logarithm of the hierarchical win, the accuracy on the coarsest aggregates reaches that attained when optimizing the coarse classification directly.

### LSHTC1

Table 6 reports results on a subset of the LSHTC1 dataset introduced by [18], which is available for download at http://lshtc.iit.demokritos.gr (specifically, “DMOZ large” for “LSHTC1”). The subset considered consists of the subtree for class 3261; this subtree includes 18 coarsest classes (though 3 of these have no corresponding samples in the testing or training sets) and 364 finest-level classes (with 288 of these having corresponding samples in the testing and training sets). We reserved one sample per finest-level class for training; all other samples were for testing, and we chose 2,000 of these uniformly at random to form the testing set. Each sample from the dataset consists of normalized, tokenized text in extracts from Wikipedia, the popular crowdsourced online encyclopedia. The hierarchy has 18 coarsest classes (with 15 actually represented in the training and testing sets), as well as 111 parents of the 288 leaves that were used in the training and testing sets. We embedded the words and their bigrams into a 1,000-dimensional space. For training the classifier into only the coarsest (aggregated) classes, we embedded the words and bigrams into a 20-dimensional space.

**Table 6:** Results on a subset of LSHTC1, tested on 2,000 samples.

training loss (rate, epochs)	one-hot win via hierarchy	softmax win via hierarchy	−log of win via hierarchy	cross entropy
flat (6, 1000)	.43	.36	1.6	5.0
raw (20000, 1000)	.23	.23	.35	∞
log (15, 15)	.36	.30	1.7	5.5
the ideal	higher	higher	lower	lower

training loss (rate, epochs)	coarsest accuracy	parents’ accuracy	finest accuracy
flat (6, 1000)	.61	.33	.23
raw (20000, 1000)	.46	.24	.01
log (15, 15)	.66	.12	.01
coarse (5, 10000)	.69		
the ideal	higher	higher	higher

training loss (rate, epochs)	aggregate precision	aggregate recall	aggregate F1
flat (6, 1000)	.38	.39	.38
raw (20000, 1000)	.23	.18	.20
log (15, 15)	.26	.29	.28
the ideal	higher	higher	higher

For this dataset, optimizing based on the hierarchical loss (with or without a logarithm) yields worse accuracy according to all metrics except the accuracy on the coarsest aggregates, compared to optimizing based on the standard cross-entropy loss. When optimizing based on the negative of the natural logarithm of the hierarchical win, the accuracy on the coarsest aggregates approaches its maximum attained when optimizing the coarse classification directly.

## Conclusion

In our experiments, optimizing the hierarchical loss (or, rather, the negative of the logarithm of the hierarchical win) using plain stochastic gradient descent with backpropagation could be helpful relative to optimizing the usual cross-entropy loss. The benefit arose mainly when there were at most a few training samples per class (of course, the training set can still be big, if there are many classes). This may help with “personalization,” which often involves limited data per individual class (even though there may be many, many individuals).

The experiments reported in Section *Results and discussion* above may be summarized as follows: relative to training on the usual cross-entropy loss, training on the negative of the logarithm of the hierarchical win hurt in all respects in Table 1, helped in all respects in Table 2, improved coarse accuracy as much as optimizing directly for coarse classification in Table 5, hurt in most respects in Table 6 while improving coarse accuracy nearly as much as optimizing directly for coarse classification, and made essentially no difference in Tables 3 and 4. Thus, whether optimizing with a hierarchical loss makes sense depends on the dataset and associated hierarchy; developing precise criteria for when the optimization with the hierarchical loss helps might be interesting.

Even so, optimizing hierarchical loss using plain stochastic gradient descent with backpropagation (as we did) is rather ineffective, at least relative to what might be possible. We trained using stochastic gradient descent with a random starting point, which may be prone to getting stuck in local optima. To some extent, hierarchical loss collapses the many classes in the hierarchy into a few aggregate superclasses, and the parameters being optimized within the aggregates should be tied closely together during the optimization—plain stochastic gradient descent is unlikely to discover the benefits of such tying, as plain stochastic gradient descent does not tie together these parameters in any way, optimizing all of them independently. Optimizing the hierarchical loss would presumably be more effective using a hierarchical process for the optimization. The hierarchical optimization could alter stochastic gradient descent explicitly into a hierarchical process, or could involve regularization terms penalizing variance in the parameters associated with the leaves in the same coarse aggregate. Unfortunately, either approach would complicate (perhaps prohibitively much) deployment to complex real-world systems. For the time being, hierarchical loss is most useful as a metric of success, gauging the performance of a fully trained classifier as a semantically meaningful figure of merit. While the performance of the hierarchical loss as an objective for optimization varies across datasets, the hierarchical loss provides a meaningful metric of success for classification on any dataset endowed with a hierarchy.

## Appendix: Minutiae of tuning hyperparameters

Table 7 elaborates on the aforementioned limited grid search performed to ascertain reasonably optimal learning rates and numbers of epochs for training.

**Table 7:** Hyperparameters for grid search.

dataset	loss	learning rates	epochs
RCV1-v2	flat	1, 2, 4	3, 4, 5, 6
RCV1-v2	raw	10, 12, 16	3, 4, 5, 6
RCV1-v2	log	2, 4, 6	3, 4, 5, 6
RCV1-v2	coarse	.03, .05, .07	50, 100, 200
Subsampled RCV1-v2	flat	.02, .03, .04, .05	8, 9, 10, 11
Subsampled RCV1-v2	raw	40, 45, 50	300, 500, 600
Subsampled RCV1-v2	log	1, 2, 3, 4	30, 40, 50
Subsampled RCV1-v2	coarse	2, 4, 8	500, 1000, 2000
Yahoo Answers	flat	.05, .1, .2, .4	2, 4, 8, 16
Yahoo Answers	raw	.5, 1, 1.5	2, 4, 8, 16
Yahoo Answers	log	.05, .1, .2	2, 4, 8, 16
Yahoo Answers	coarse	.05, .1, .2	2, 4, 8
DBpedia	flat	.125, .25, .5, 1, 2	2, 4, 8
DBpedia	raw	.5, 1, 2, 4	2, 4, 8
DBpedia	log	.125, .25, .5, 1, 2	2, 4, 8
DBpedia	coarse	.1, .2, .3, .4	2, 4, 8
DBpedia fish	flat	3, 5, 10	20, 25, 30
DBpedia fish	raw	100, 200, 300	100, 200, 300
DBpedia fish	log	3, 5, 10	25, 50, 100
DBpedia fish	coarse	1, 2, 3, 4	50, 60, 70, 80
LSHTC1	flat	2, 4, 6, 8	500, 1000, 2000
LSHTC1	raw	10000, 20000, 30000	500, 1000, 2000
LSHTC1	log	10, 15, 20	10, 15, 20
LSHTC1	coarse	3, 5, 7	5000, 10000, 20000

## References

[1] LeCun Y, Bengio Y, Hinton G. Deep learning. Nature. 2015;521(7553):436–444. 10.1038/nature14539 26017442

[2] Cai L, Hofmann T. Hierarchical document categorization with support vector machines. In: Proc. 13th ACM Internat. Conf. Information and Knowledge Management. ACM; 2004. p. 78–87.

[3] Kosmopoulos A, Partalas I, Gaussier E, Paliouras G, Androutsopoulos I. Evaluation measures for hierarchical classification: a unified view and novel approaches. Data Mining and Knowledge Discovery. 2015;29(3):820–865. 10.1007/s10618-014-0382-x

[4] Binder A, Kawanabe M, Brefeld U. Efficient classification of images with taxonomies. In: Proc. 9th Asian Conf. Computer Vision. vol. 5996 of Lecture Notes in Computer Science. Springer; 2009. p. 351–362.

[5] Chang JY, Lee KM. Large margin learning of hierarchical semantic similarity for image classification. Computer Vision and Image Understanding. 2015;132:3–11. 10.1007/s11263-014-0790-9

[6] Costa EP, Lorena AC, Carvalho ACPLF, Freitas AA. A review of performance evaluation measures for hierarchical classifiers. In: Drummond C, Elazmeh W, Japkowicz N, Macskassy SA, editors. Evaluation Methods for Machine Learning II: Papers from the AAAI-2007 Workshop. AAAI Press; 2007. p. 182–196.

[7] Deng J, Berg AC, Li K, Li FF. What does classifying more than 10,000 image categories tell us? In: Proc. 11th European Conf. Computer Vision. vol. 5. Springer-Verlag; 2010. p. 71–84.

[8] Deng J, Berg AC, Li K, Li FF. Hierarchical semantic indexing for large scale image retrieval. In: Proc. IEEE Conf. Computer Vision and Pattern Recognition. IEEE; 2011. p. 785–792.

[9] Wang K, Zhou S, Liew SC. Building hierarchical classifiers using class proximity. In: Proc. 25th Internat. Conf. Very Large Data Bases. Morgan Kaufmann Publishers; 1999. p. 363–374.

[10] Reece JB, Urry LA, Cain ML, Wasserman SA, Minorsky PV, Jackson RB. Campbell Biology. 10th ed Pearson; 2013.

[11] Silla CN Jr, Freitas AA. A survey of hierarchical classification across different application domains. J. Data Mining Knowledge Discovery. 2011;22(1–2):31–72. 10.1007/s10618-010-0175-9

[12] Kosmopoulos A, Paliouras G, Androutsopoulos I. Probabilistic cascading for large-scale hierarchical classification. arXiv; 2015. 1505.02251. Available from: http://arxiv.org/abs/1505.02251.

[13] Redmon J, Farhadi A. YOLO9000: better, faster, stronger. In: IEEE Conf. Comput. Vision Pattern Recognition. IEEE; 2017. p. 1–9.

[14] Joulin A, Grave E, Bojanowski P, Mikolov T. Bag of tricks for efficient text classification. In: Proc. 15th Conf. European Chapter Assoc. Comput. Linguistics. ACL; 2017. p. 427–431.

[15] Lewis DD, Yang Y, Rose TG, Li F. RCV1: a new benchmark collection for text categorization research. J. Machine Learning Research. 2004;5:361–397.

[16] Zhang X, Zhao J, LeCun Y. Character-level convolutional networks for text classification In: Advances in Neural Information Processing Systems. vol. 28 Neural Information Processing Systems Foundation; 2015 p. 1–9.

[17] Lehmann J, Isele R, Jakob M, Jentzsch A, Kontokostas D, Mendes PN, et al DBpedia—a large-scale, multilingual knowledge base extracted from Wikipedia. Semantic Web. 2015;6(2):167–195. 10.3233/SW-140134

[18] Partalas I, Kosmopoulos A, Baskiotis N, Artieres T, Paliouras G, Gaussier E, et al. LSHTC: a benchmark for large-scale text classification. arXiv; 2015. 1503.08581. Available from: http://arxiv.org/abs/1503.08581.

